# Supplementary material for: In situ continuous Dopa supply by responsive artificial enzyme for the treatment of Parkinson’s disease
Source: Nat Commun. 2023 May 9;14:2661. doi: 10.1038/s41467-023-38323-w (PMC10169781; doi:10.1038/s41467-023-38323-w)
Supplement: Supplementary file 1 — Supplementary Information [file 41467_2023_38323_MOESM1_ESM.pdf]

Supplementary Information

In situ continuous Dopa supply by responsive artificial enzyme for the treatment of Parkinson's disease

Xiao Fang[1], Meng Yuan[1], Fang Zhao[1], Aoling Yu[1], Qianying Lin[1], Shiqing Li[1], Huichen Li[1], Xinyang Wang[1], Yanbin Yu[1], Xin Wang[1], Qitian Lin[1], Chunhua Lu[1,]*, Huanghao Yang[1,]*

[1]MOE Key Laboratory for Analytical Science of Food Safety and Biology, Fujian Provincial Key Laboratory of Analysis and Detection Technology for Food Safety, State Key Laboratory of Photocatalysis on Energy and Environment, College of Chemistry, Fuzhou University, Fuzhou 350108, P. R. China.

*E-mail: chunhualu@fzu.edu.cn

hhyang@fzu.edu.cn

Supplementary Tables

Supplementary Table 1. Nucleic acid sequences used in this study.

In the primary sequences listed below, the tyrosine aptamer is in red, the TfR aptamer is in green, the ASO is in orange.

Name		Detailed sequences information
Apt-Fe_3O_4	Tyrosine aptamer	5'-(NH_2 C6)-TGTATGTGGTGTGTGAGTGCGGTGCCCAGTGTTC-3'
Cy5-labeled tyrosine aptamer complementary strand		5'-GCGTGGTCACACGCGAGCCTACATAGAGAACACT(Cy5)GGGCA-3'
Ran-Fe_3O_4	Random sequence	5'-(NH_2 C6)-TGTAAGGCCGGTTGGCTGTGTGTGCCCAGTGTTC-3'
FNA-Fe_3O_4	Tyrosine aptamer	5'-(NH_2 C6)-TGTATGTGGTGTGTGAGTGCGGTGCCCAGT(BHQ2)GTTC-3'
	Block strand	5'-GCGTGGTCACACGCGAGCCTACATAGAGAACACT(Cy5)GGGCA-3'
Fluorescence-on Apt-Fe_3O_4	Tyrosine aptamer	5'-(NH_2 C6)-TGTATGTGGTGTGTGAGTGCGGTGCCCAGT(Cy5)GTTC-3'
Fluorescence-on Ran-Fe_3O_4	Random sequence	5'-(NH_2 C6)-TGTAAGGCCGGTTGGCTGTGTGTGCCCAGT(Cy5)GTTC-3'
Fluorescence-on FNA -Fe_3O_4	Tyrosine aptamer	5'-(NH_2 C6)-TGTATGTGGTGTGTGAGTGCGGTGCCCAGT(Cy5)GTTC-3'
	Block strand	5'-GCGTGGTCACACGCGAGCCTACATAGAGAACACTGGGCA-3'
Cat-Fe_3O_4	Tyrosine aptamer	5'-(NH_2 C6)-TGTATGTGGTGTGTGAGTGCGGTGCCCAGTGTTCAGCCAA -3'
	Block strand	5'-GCGTGGTCACACGCAGGCCGGTTGGCTGAACAC-3'
ASO-Fe_3O_4	Random sequence	5'-(NH_2 C6)-TGTAAGGCCGGTTGGCTGTGTGTGCCCAGTGTTC-3'
	Block strand	5'-GCGTGGTCACACGCGAGCCTACATAGAGAACACTGGGCA-3'
Targeted region of SNCA mRNA		5'-GAGGGTGTTCTCTATGTAGGCTC-3'
SNCB mRNA		5'-GAGGGCGTCCTCTACGTCGGAAG-3'
SNCG mRNA		5'-GAGGGGGTCATGTATGTGGGAGC-3'
mouse SNCA mRNA		5'-GAGGGAGTCCTCTATGTAGGTTC-3'

Supplementary Table 2. Kinetic parameters associated with the artificial enzyme and control nanomaterials[a].

		V_{max} (μM min^{-1})	K_M (μM)
In aqueous solution	Fe_3O_4	4.62 ± 0.10	1540 ± 60
	Ran-Fe_3O_4	4.84 ± 0.10	1656 ± 59
	Apt-Fe_3O_4	6.47 ± 0.26	444 ± 48
	FNA-Fe_3O_4	5.33 ± 0.28	1288 ± 128
	FNA-Fe_3O_4 + mRNA	6.08 ± 0.15	491 ± 31
In crowded solution	Fe_3O_4	3.92 ± 0.82	5601 ± 1503
	Ran-Fe_3O_4	3.19 ± 0.28	3762 ± 458
	Apt-Fe_3O_4	5.97 ± 0.18	564 ± 44
	FNA-Fe_3O_4	4.79 ± 0.10	3475 ± 98
	FNA-Fe_3O_4 + mRNA	5.58 ± 0.18	639 ± 51

[a]All experiments were performed in a 10 mM Tris-HCl buffer solution, pH 7.2, that included 1 mM $MgCl_2$, 50 mM NaCl, and 10 μg·mL^{-1} of the respective artificial enzymes or control nanomaterials and 5 mM H_2O_2, 5 mM AA.

Supplementary Figures

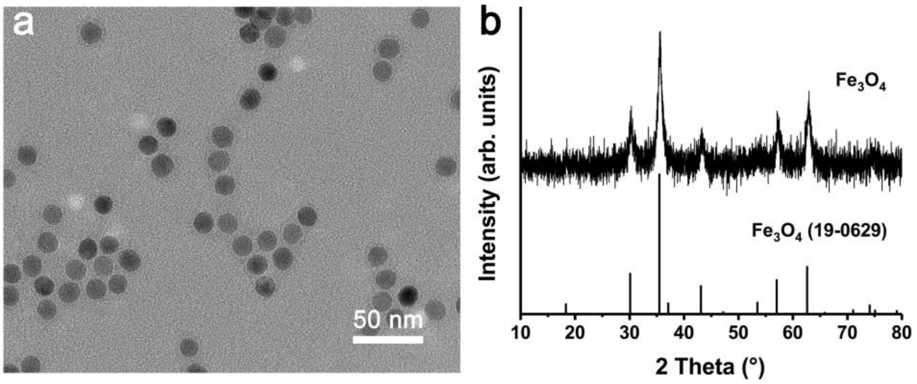

Supplementary Fig. 1: Characterizations of Fe_3O_4 nanoparticles. **a**, TEM image of Fe_3O_4 nanoparticles. **b**, XRD pattern of Fe_3O_4 nanoparticles. **a**, one representative data was shown from three independently repeated experiments. Source data are provided as a Source Data file.

Fe_3O_4 nanoparticles exhibit a spherical shape with average dimension of 15 nm (Supplementary Fig. 1a). The XRD pattern of Fe_3O_4 showed the typical bands for magnetite with a cubic spinel structure, space group *Fd*3*m* (227) (Supplementary Fig. 1b).

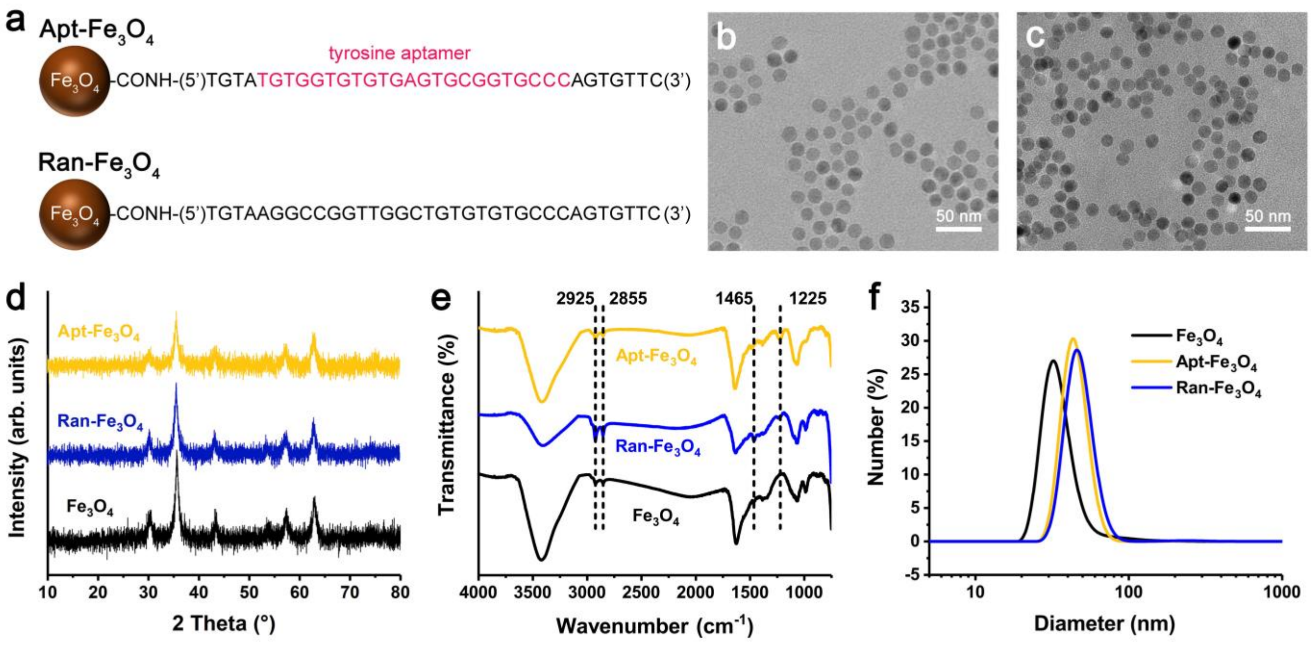

Supplementary Fig. 2: Characterizations of Apt-Fe_3O_4 and Ran-Fe_3O_4. **a**, DNA sequences in Apt-Fe_3O_4 and Ran-Fe_3O_4. **b**, **c**, TEM images of Apt-Fe_3O_4 (**b**) and Ran-Fe_3O_4 (**c**). **d**, XRD patterns of Fe_3O_4, Ran-Fe_3O_4 and Apt-Fe_3O_4. **e**, FT-IR spectra of Fe_3O_4, Ran-Fe_3O_4 and Apt-Fe_3O_4. **f**, Hydrodynamic sizes of Fe_3O_4, Ran-Fe_3O_4 and Apt-Fe_3O_4. **b**, **c**, one representative data was shown from three independently repeated experiments. Source data are provided as a Source Data file.

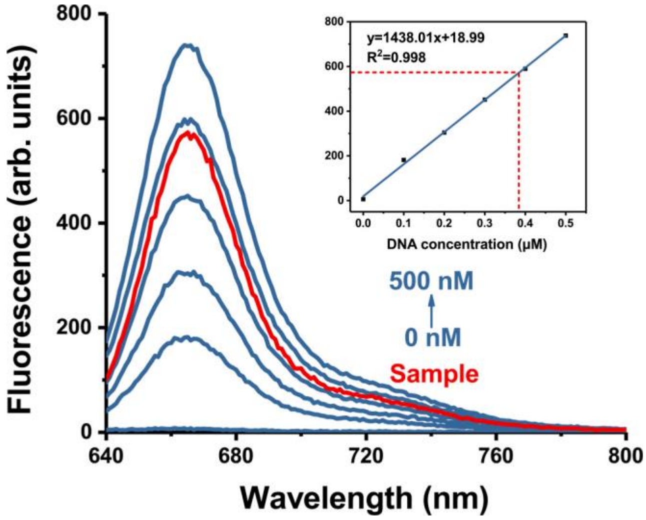

Supplementary Fig. 3: The amount of tyrosine aptamer loaded on Apt-Fe_3O_4 via amide bonds. Source data are provided as a Source Data file.

25 μg of Apt-Fe_3O_4 was hybridized with 0.5 nmol of Cy5-labeled tyrosine aptamer complementary strand (sequence: 5'-(6-FAM)-GACCACTCACCTACCGTTTGCCCTGGTC-3') in 1 ml PBS (pH=7.4) at 37 °C for 2 h. The reaction solution was then magnetic separated, and the Cy5 fluorescence spectrum of the supernatant was measured. The standard curve of the Cy5-labeled tyrosine aptamer complementary strand was determined by the fluorescence intensity at 665 nm, and the amount of tyrosine aptamer loaded on Apt-Fe_3O_4 was calculated as 4.59 μmol g^{-1}.

The radius of each Fe_3O_4 core (R) was regarded as 7.5 nm. The number of aptamer strands on each nanoparticle (n) was calculated as $n=(4/3)\pi R^3\rho(Fe_3O_4)*4.59*10^{-6}N_A=25$.

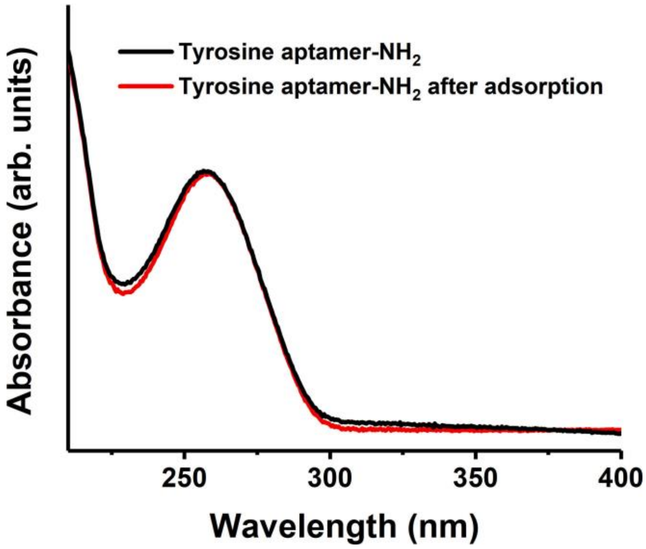

Supplementary Fig. 4: The UV-vis absorption spectra of supernatants of 5'-terminal amino-modified tyrosine aptamer before and after being adsorbed by Fe_3O_4 via nonspecific interactions. Source data are provided as a Source Data file.

Nonspecific adsorption of DNA strands on Fe_3O_4 was confirmed to be almost absent by UV-vis absorption spectroscopic analysis.

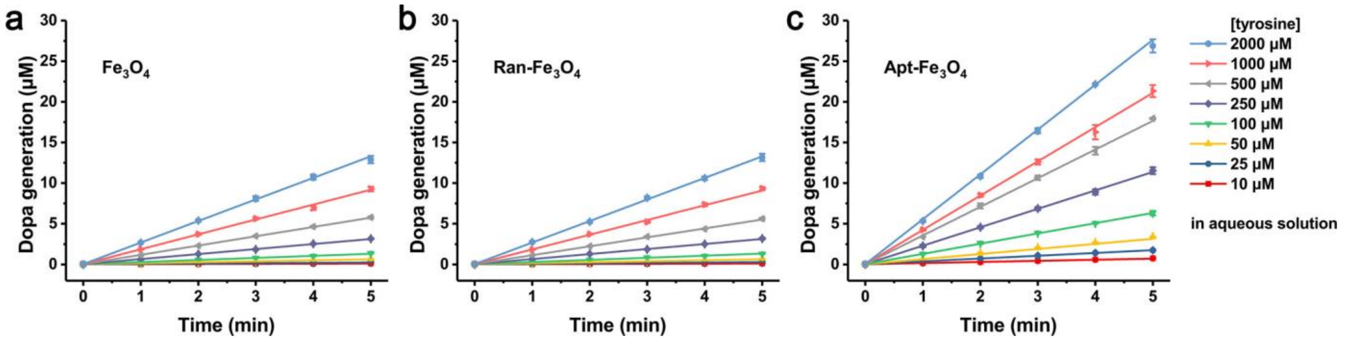

Supplementary Fig. 5: Time-dependent Dopa generation upon the hydroxylation of different concentrations of tyrosine (10, 25, 50, 100, 250, 500, 1000, 2000 μM) by (a) Fe_3O_4; (b) Ran-Fe_3O_4; (c) Apt-Fe_3O_4 in aqueous solution. The results were expressed as mean ± SD (*n* = 3 independent experiments). Source data are provided as a Source Data file.

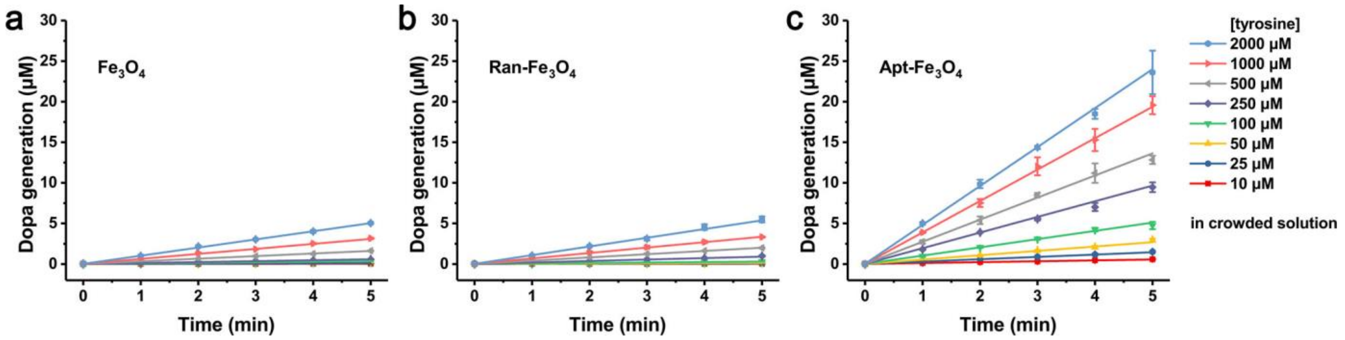

Supplementary Fig. 6: Time-dependent Dopa generation upon the hydroxylation of different concentrations of tyrosine (10, 25, 50, 100, 250, 500, 1000, 2000 μM) by (a) Fe_3O_4; (b) Ran-Fe_3O_4; (c) Apt-Fe_3O_4 in crowded 20 wt% PEG-20000 solution. The results were expressed as mean ± SD (*n* = 3 independent experiments). Source data are provided as a Source Data file.

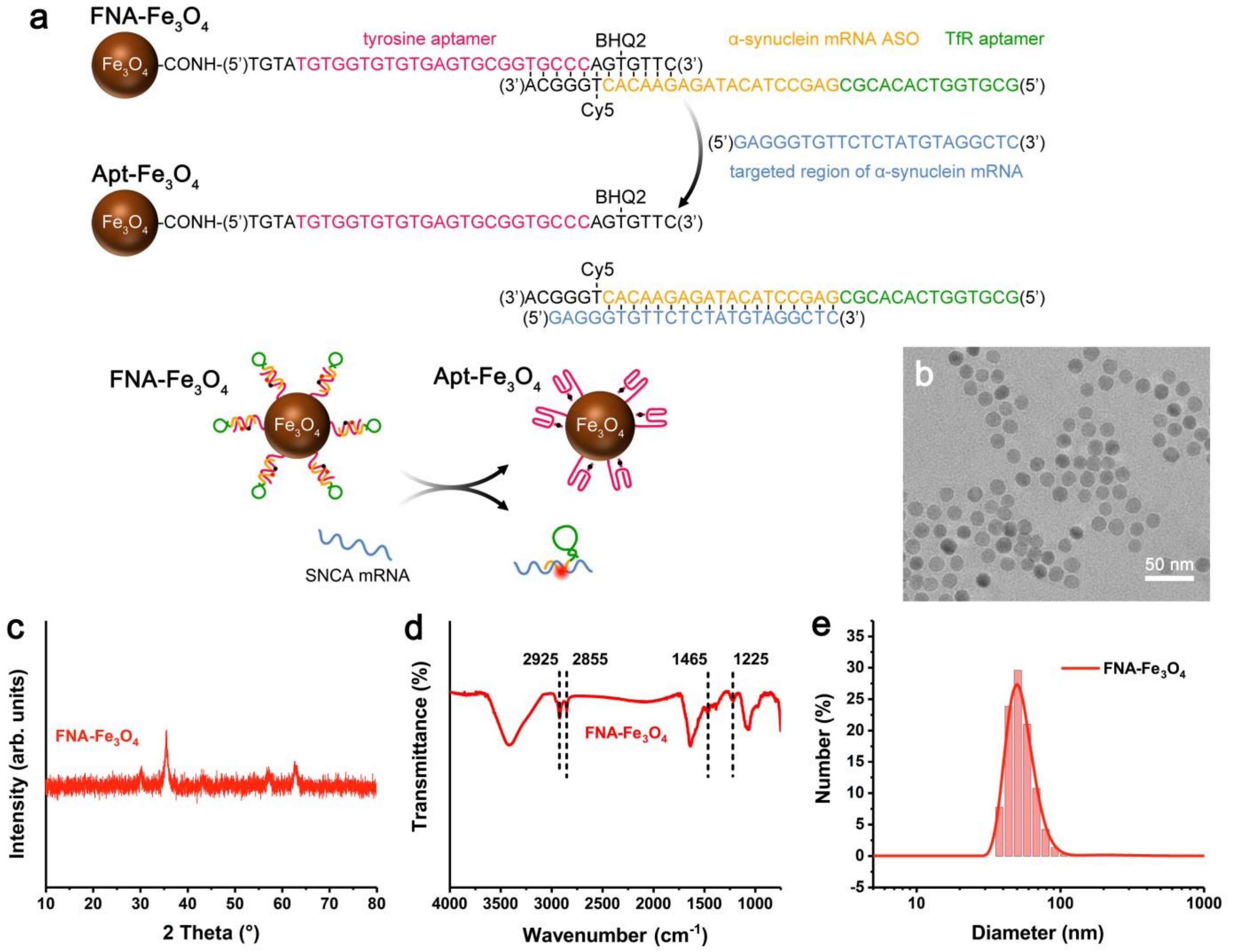

Supplementary Fig. 7: Characterizations of FNA-Fe_3O_4. a, Functional nucleic acids structure of FNA-Fe_3O_4. **b**, TEM image of FNA-Fe_3O_4. **c**, XRD pattern of FNA-Fe_3O_4. **d**, FT-IR spectrum of FNA-Fe_3O_4. **e**, Hydrodynamic size of FNA-Fe_3O_4. **b**, one representative data was shown from three independently repeated experiments. Source data are provided as a Source Data file.

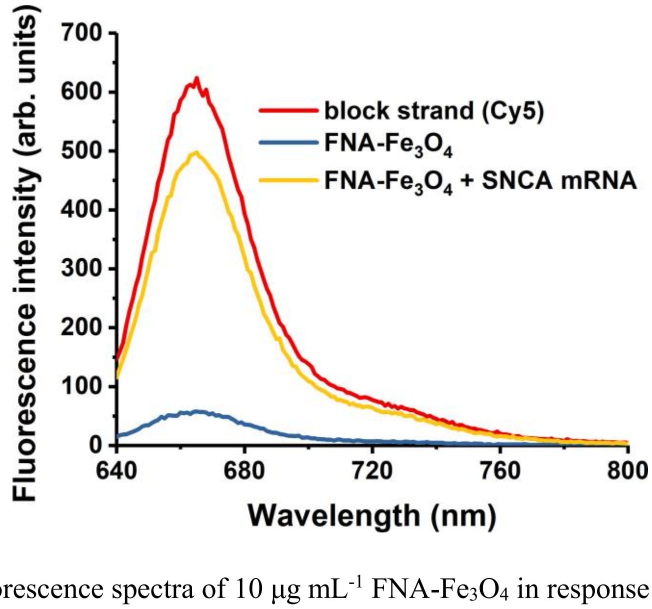

Supplementary Fig. 8: Fluorescence spectra of 10 μg mL^{-1} FNA-Fe_3O_4 in response to SNCA mRNA (30 nM). Source data are provided as a Source Data file.

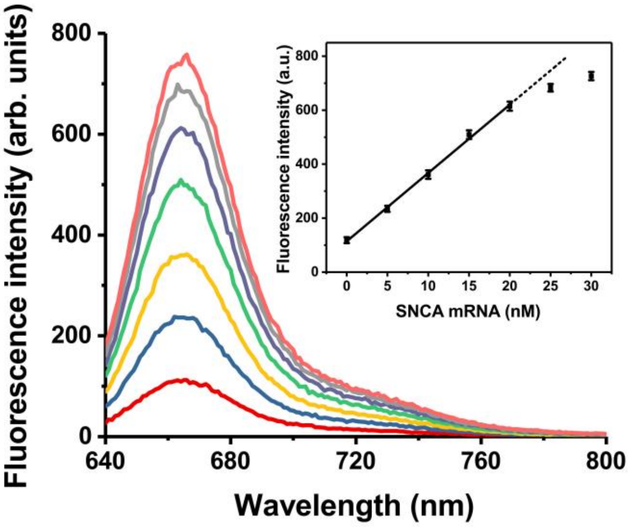

Supplementary Fig. 9: Fluorescence spectra of 10 µg mL^{-1} FNA-Fe_3O_4 incubated with SNCA mRNA at varying concentrations, one representative data was shown from three independently repeated experiments; The inset shows the linear relationship between fluorescence intensity and SNCA mRNA concentration, the results were expressed as mean ± SD (n = 3 independent experiments). Source data are provided as a Source Data file.

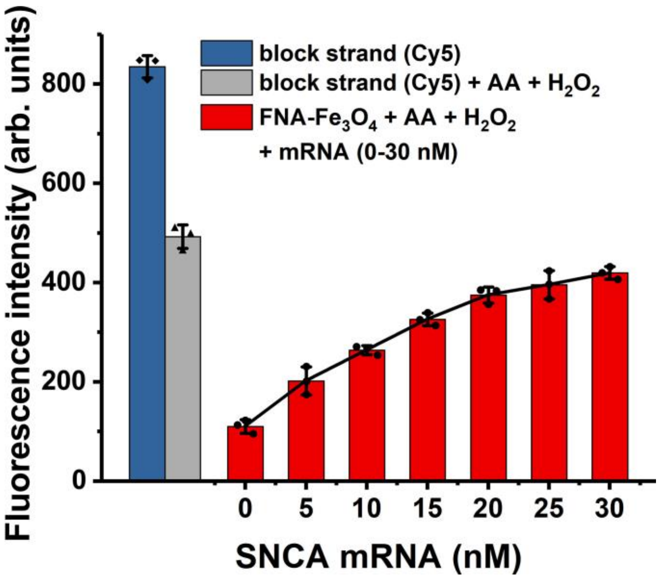

Supplementary Fig. 10: The fluorescence quenching of Cy5 and the fluorescence intensities of 10 µg mL^{-1} FNA-Fe_3O_4 incubated with SNCA mRNA at varying concentrations in the presence of 5 mM AA and 5 mM H_2O_2. The results were expressed as mean ± SD (n = 3 independent experiments). Source data are provided as a Source Data file.

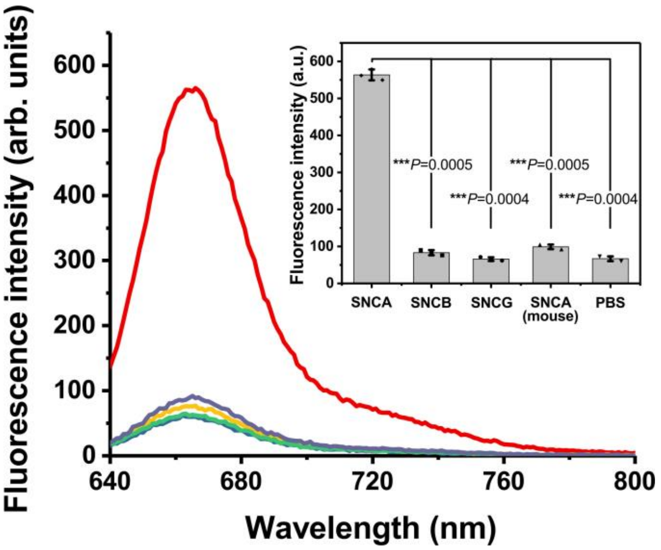

Supplementary Fig. 11: Fluorescence spectra of 10 μg mL^{-1} FNA-Fe_3O_4 incubated with different mRNAs (20 nM), one representative data was shown from three independently repeated experiments; The inset shows the corresponding fluorescence intensity, the results were expressed as mean ± SD (n = 3 independent experiments). P-values were calculated by two-tailed t-test. Source data are provided as a Source Data file.

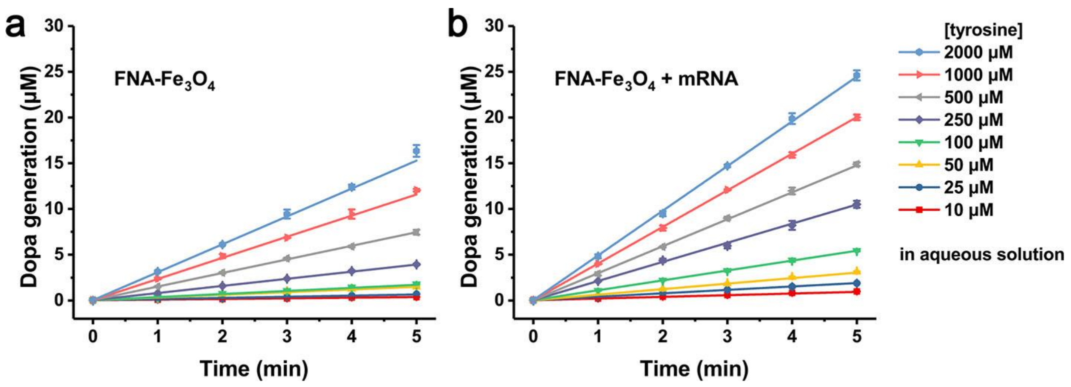

Supplementary Fig. 12: Time-dependent Dopa generation upon the hydroxylation of different concentrations of tyrosine (10, 25, 50, 100, 250, 500, 1000, 2000 μM) by (a) FNA-Fe_3O_4; (b) FNA-Fe_3O_4 + mRNA in aqueous solution. The results were expressed as mean ± SD (n = 3 independent experiments). Source data are provided as a Source Data file.

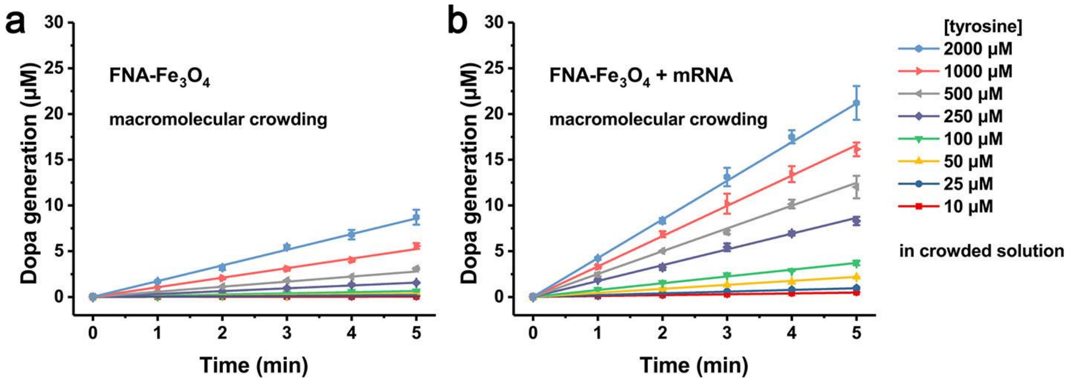

Supplementary Fig. 13: Time-dependent Dopa generation upon the hydroxylation of different concentrations of tyrosine (10, 25, 50, 100, 250, 500, 1000, 2000 μM) by (a) FNA-Fe_3O_4; (b) FNA-Fe_3O_4 + mRNA in crowded 20 wt% PEG-20000 solution. The results were expressed as mean ± SD (n = 3 independent experiments). Source data are provided as a Source Data file.

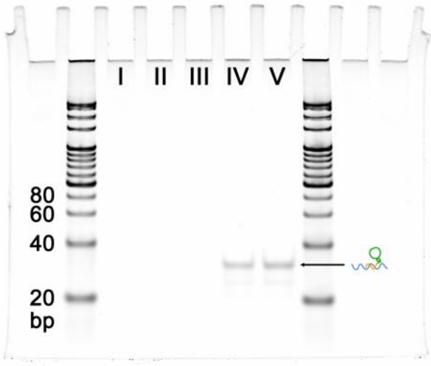

Supplementary Fig. 14: 10 μg mL^{-1} of Apt-Fe_3O_4, Ran-Fe_3O_4, FNA-Fe_3O_4, FNA-Fe_3O_4+mRNA were reacted in a mixed solution containing tyrosine (100 μM), H_2O_2 (5 mM), and ascorbic acid (5 mM) for native PAGE image after 30 min of reaction (I: Apt-Fe_3O_4, II: Ran-Fe_3O_4, III: FNA-Fe_3O_4, IV: FNA-Fe_3O_4+mRNA, V: block strand+mRNA). Gel was representative of n = 3 independent experiments.

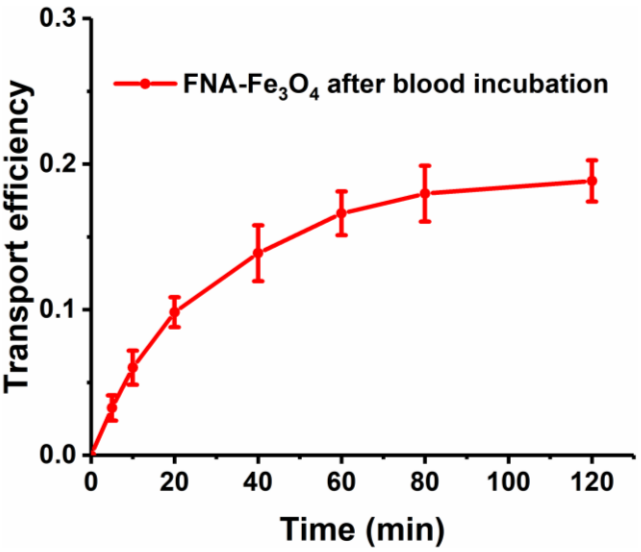

Supplementary Fig. 15: Transport efficiency of FNA-Fe_3O_4 across the BBB cell model after FNA-Fe_3O_4 incubated with blood for 4 h and magnetically separated. The results were expressed as mean ± SD (n = 3 biologically independent samples). Source data are provided as a Source Data file.

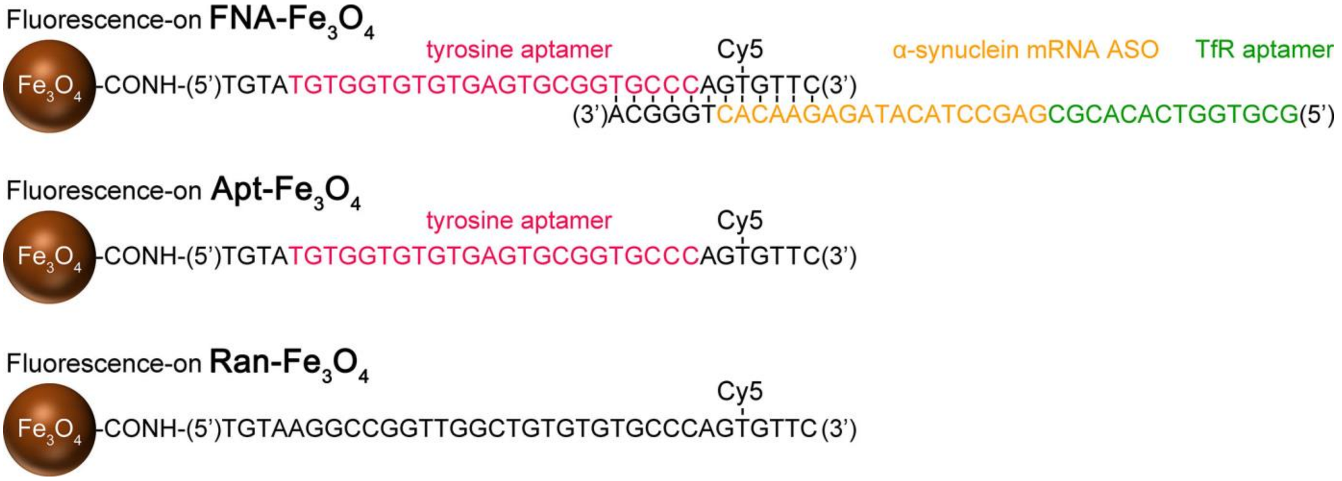

Supplementary Fig. 16: Functional nucleic acids structure of Fluorescence-on FNA-Fe_3O_4, Apt-Fe_3O_4 and Ran-Fe_3O_4.

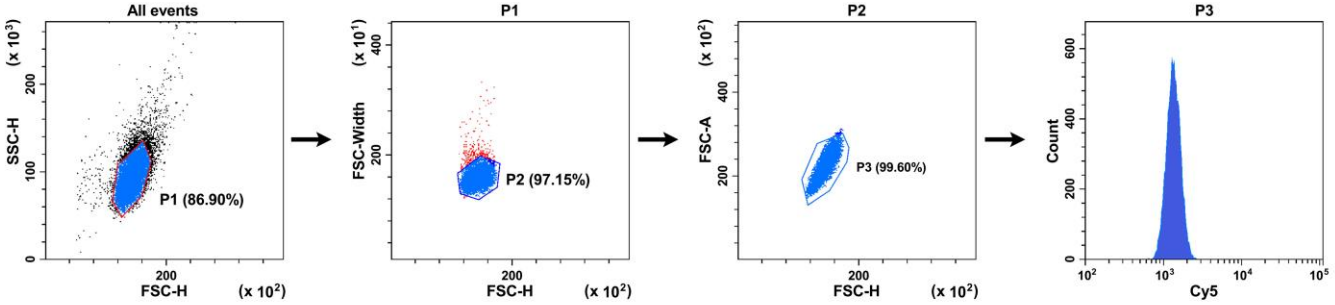

Supplementary Fig. 17: Gating strategy for analyzing cellular uptake by flow cytometry in Fig. 4c.

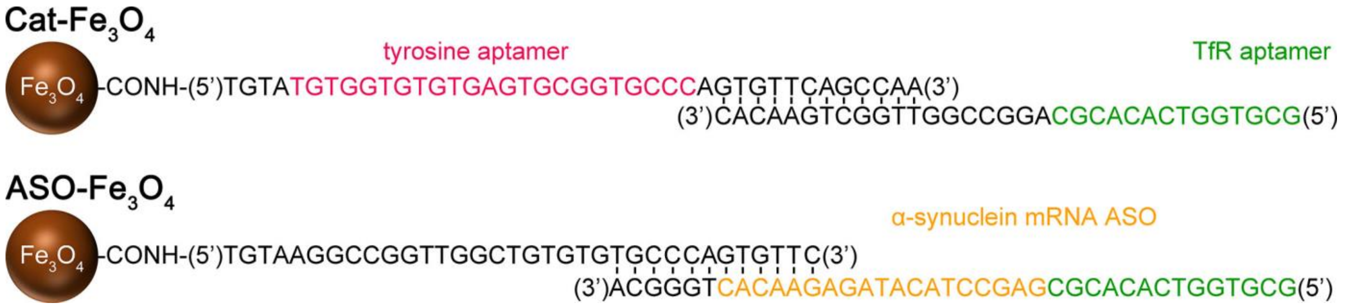

Supplementary Fig. 18: Functional nucleic acids structure of Cat-Fe_3O_4 and ASO-Fe_3O_4.

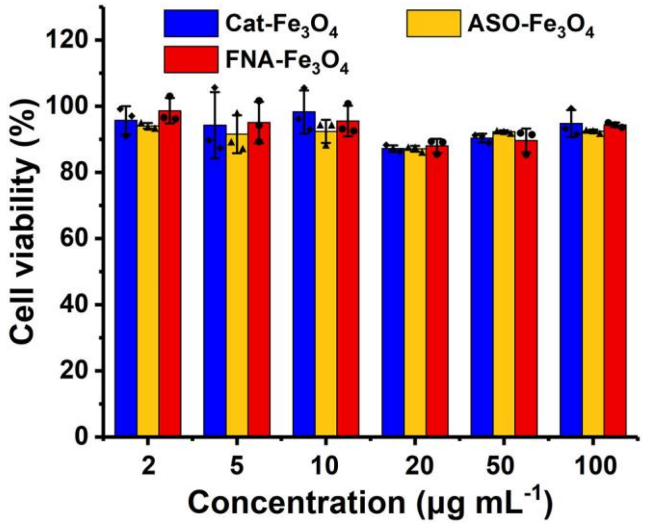

Supplementary Fig. 19: Cell viability of SH-SY5Y cells incubated with the artificial enzymes at different concentrations. The results were expressed as mean ± SD (n = 3 biologically independent samples). Source data are provided as a Source Data file.

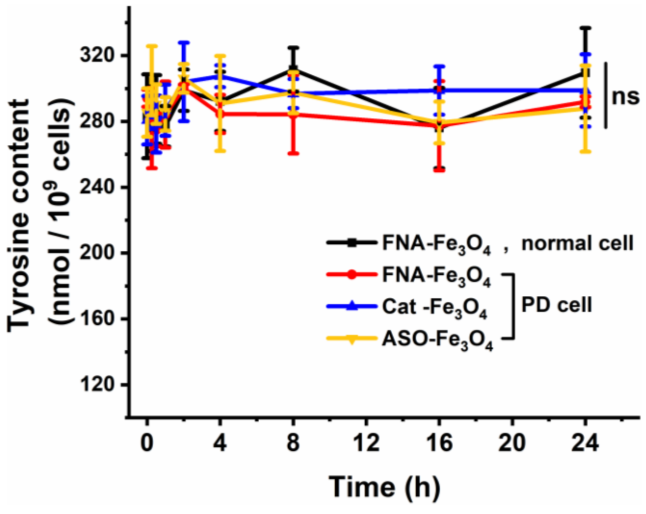

Supplementary Fig. 20: Intracellular tyrosine content in artificial enzymes treated cells. The results were expressed as mean ± SD (n = 3 biologically independent samples). Source data are provided as a Source Data file.

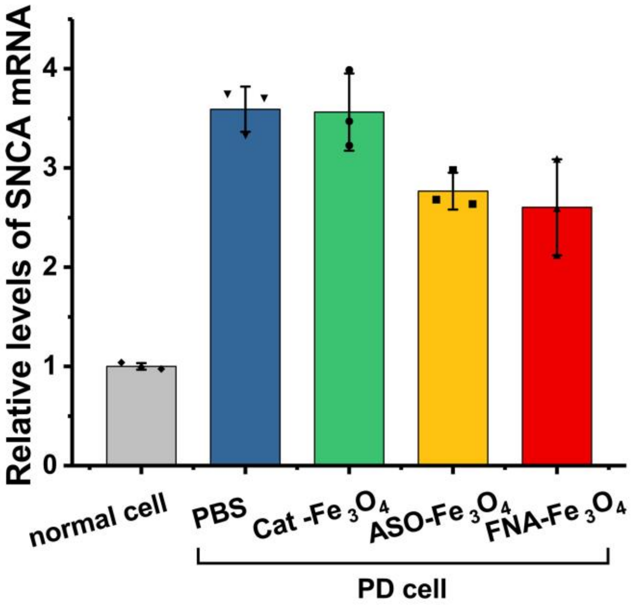

Supplementary Fig. 21: SNCA mRNA expression in diffierent treated SH-SY5Y cells. The results were expressed as mean ± SD (n = 3 biologically independent samples). Source data are provided as a Source Data file.

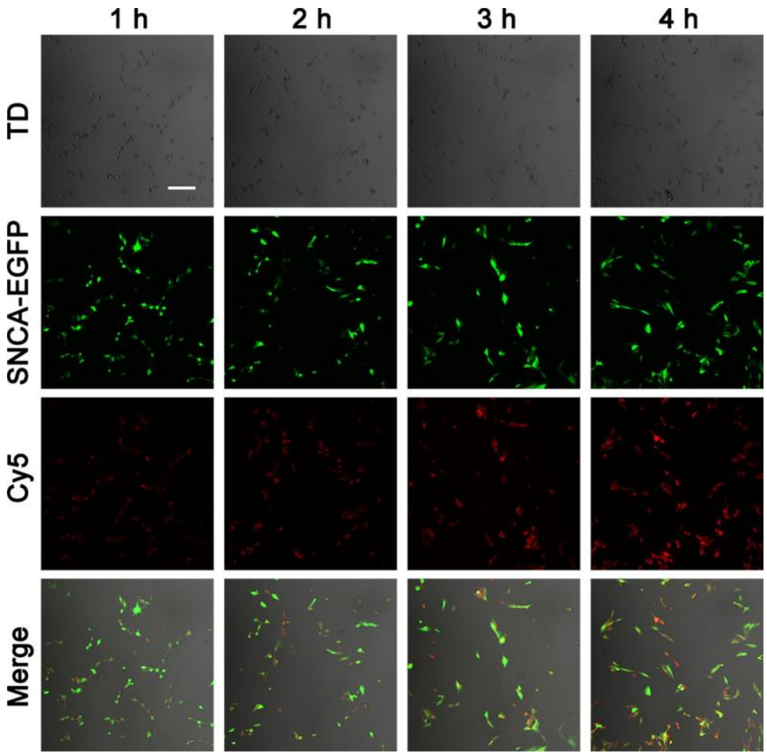

Supplementary Fig. 22: Confocal fluorescence images of SNCA miRNA in SH-SY5Y cells incubated with FNA-Fe_3O_4 (100 μg mL^{-1}) for various times (1, 2, 3, 4 h). Scale bar: 100 μm. The representative data was shown from three independently repeated experiments.

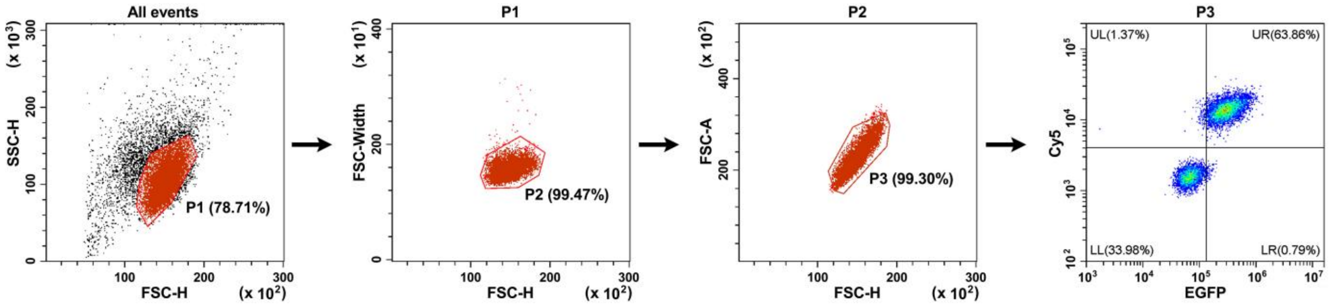

Supplementary Fig. 23: Gating strategy for isolating Cy5$^+$EGFP$^+$ SH-SY5Y cells in Fig. 5h.

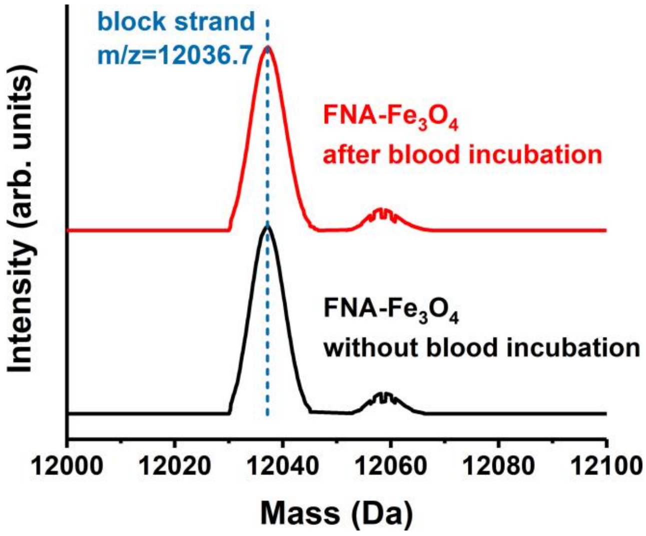

Supplementary Fig. 24: Mass spectra of the block strand in FNA-Fe_3O_4 after and without blood incubation. Peak at m/z=12036.7 represent block strand. Source data are provided as a Source Data file.

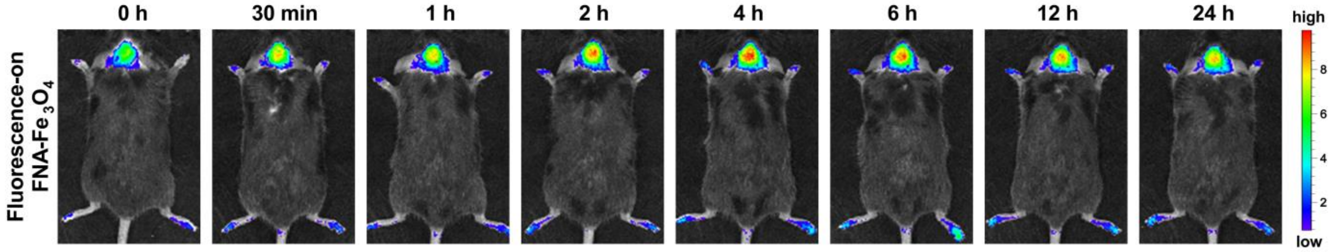

Supplementary Fig. 25: In vivo brain accumulation in mice injected with fluorescence-on FNA-Fe_3O_4 for different time periods.

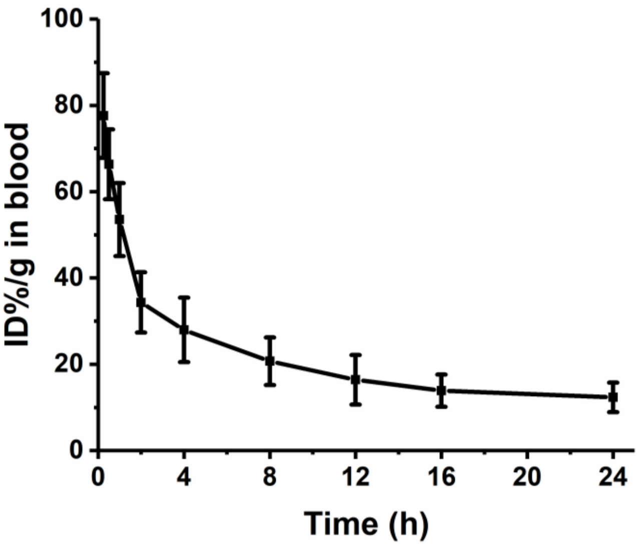

Supplementary Fig. 26: Blood circulation curve of FNA-Fe_3O_4 in vivo. The results were expressed as mean ± SD (*n* = 3 biologically independent animals). Source data are provided as a Source Data file.

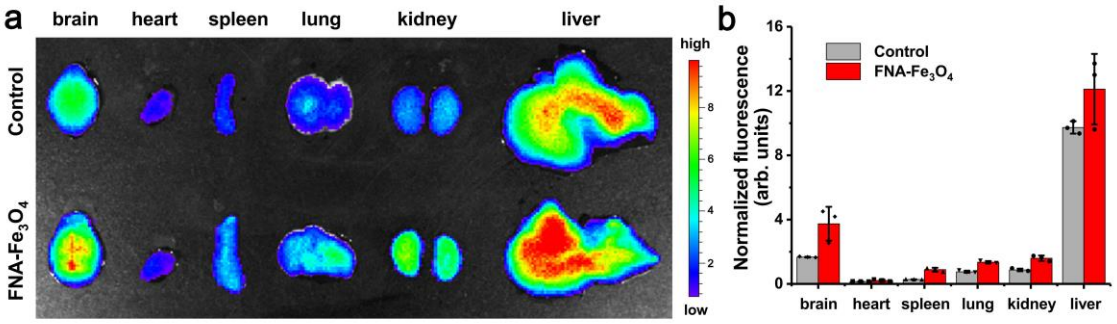

Supplementary Fig. 27: **a**, Ex vivo fluorescence organ images of mice injected with and without fluorescence-on FNA-Fe_3O_4 for 4h. **b**, Quantitative analysis of the normalized fluorescence signals of the organs in (**a**). The results were expressed as mean ± SD (*n* = 3 independent experiments). Source data are provided as a Source Data file.

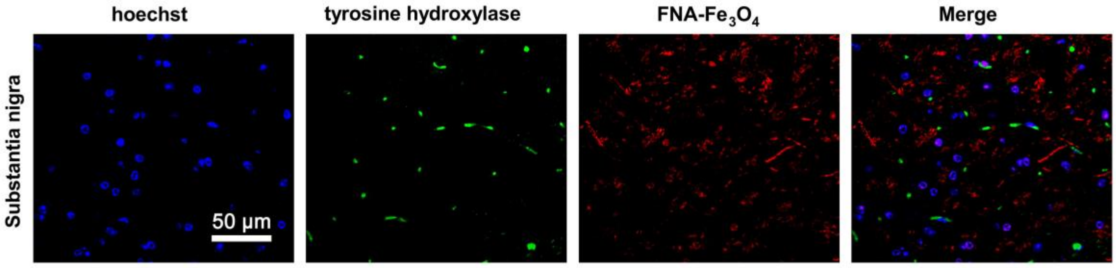

Supplementary Fig. 28: Distribution of tyrosine hydroxylase-positive neurons and FNA-Fe_3O_4 in brain slices from mice treated with fluorescence-on FNA-Fe_3O_4 for 4h. The representative data was shown from three independently repeated experiments.

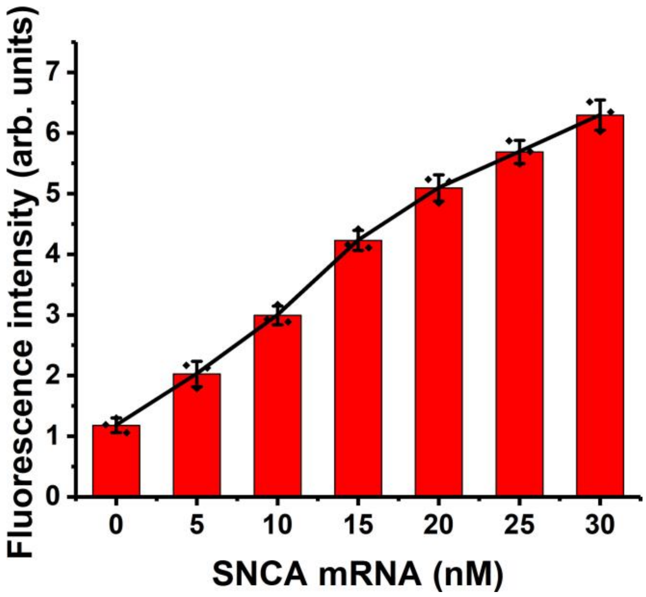

Supplementary Fig. 29: The fluorescence responsiveness of 10 μg mL^{-1} FNA-Fe_3O_4 incubated with SNCA mRNA at varying concentrations after FNA-Fe_3O_4 incubated with blood for 4 h. The results were expressed as mean ± SD (n = 3 independent experiments). Source data are provided as a Source Data file.

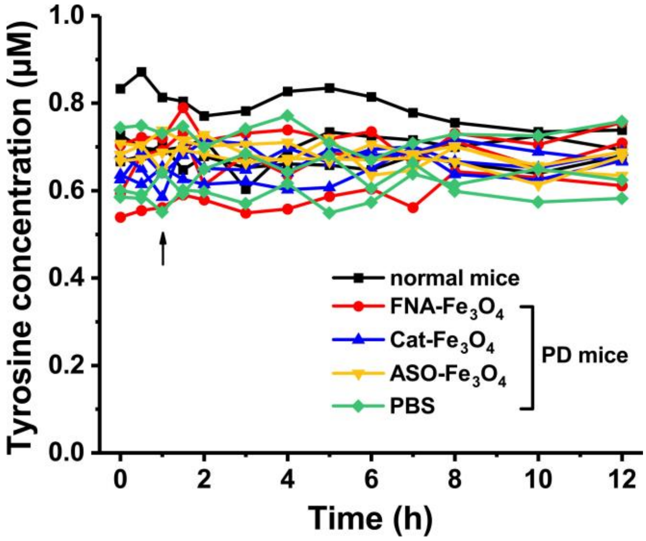

Supplementary Fig. 30: Tyrosine levels in mice striatal microdialysates under the influence of artificial enzymes (arrows represent dosing time point, each line represent one mouse, n = 3 biologically independent animals). Source data are provided as a Source Data file.

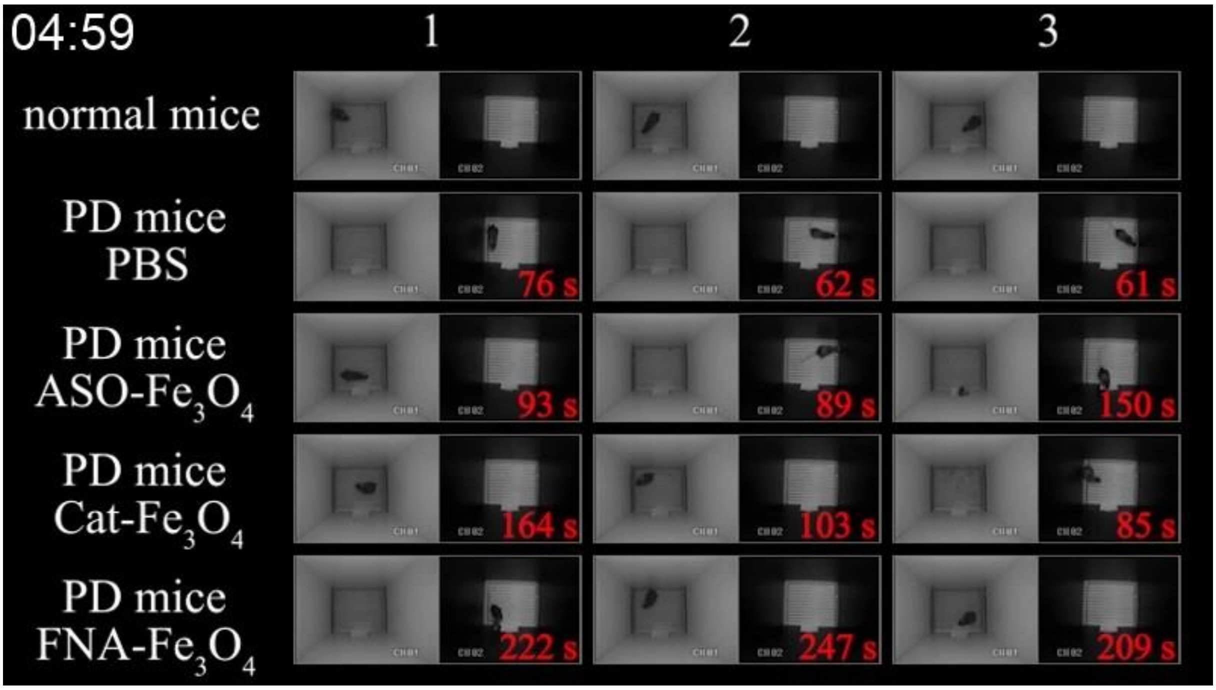

Supplementary Fig. 31: The recorded image of 300 s of the video in the step-through passive avoidance test. The time record in red indicated the step-through latency time of mice (each panel represent one mouse, n = 3 biologically independent animals).

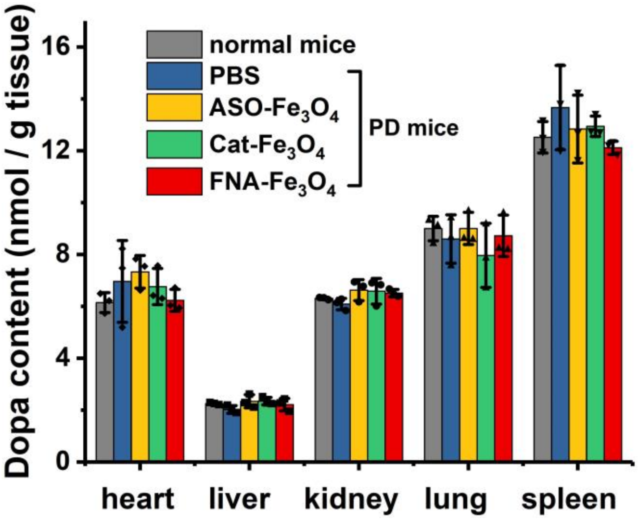

Supplementary Fig. 32: Dopa content in peripheral tissues of normal mice, PD mice, and artificial enzymes treated PD mice. The results were expressed as mean ± SD (n = 3 biologically independent animals). Source data are provided as a Source Data file.

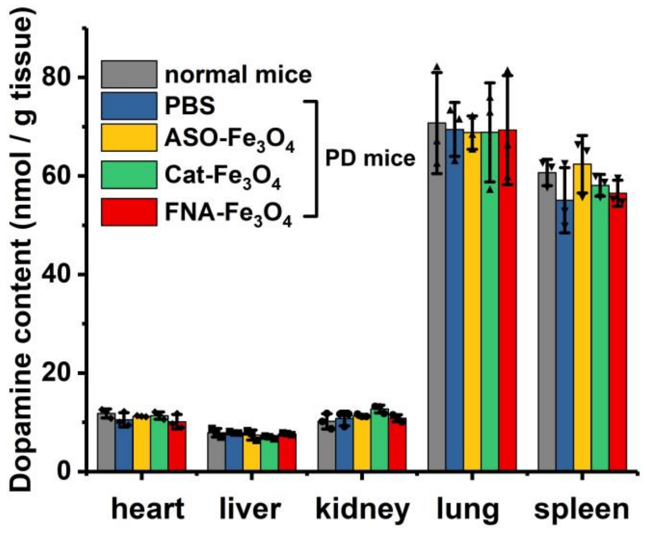

Supplementary Fig. 33: Dopamine content in peripheral tissues of normal mice, PD mice, and artificial enzymes treated PD mice. The results were expressed as mean ± SD ($n = 3$ biologically independent animals). Source data are provided as a Source Data file.

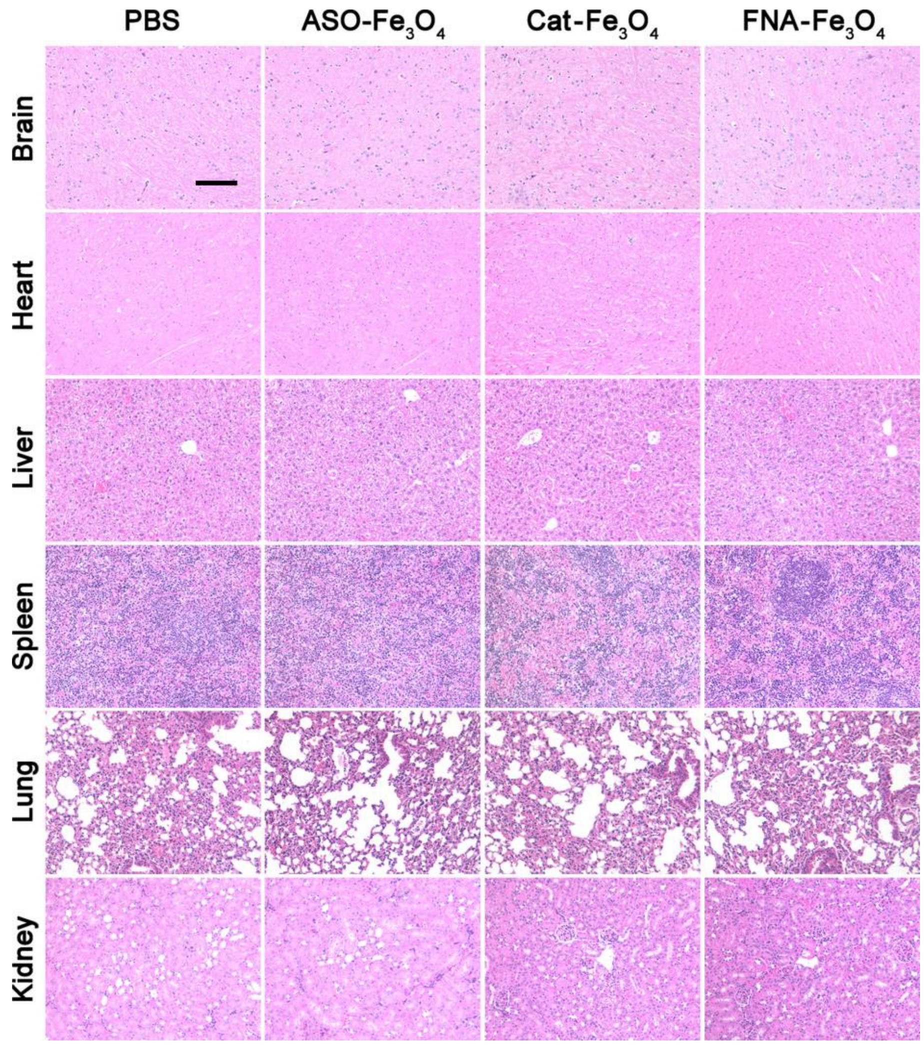

Supplementary Fig. 34: H&E stained images of the main organs of each group of mice. Scale bar: 200 μm. The representative data was shown from three independently repeated experiments.

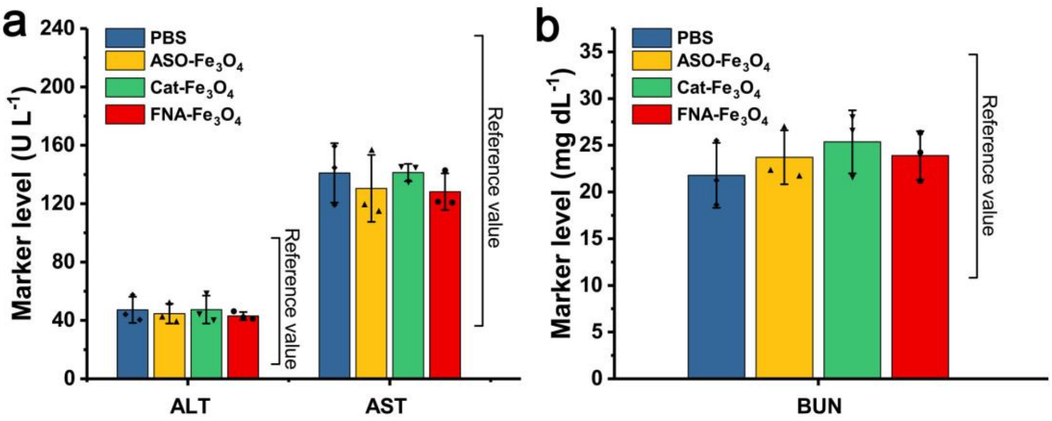

Supplementary Fig. 35: **a**, **b**, Serum biochemistry assays of liver function markers ALT, AST (**a**) and kidney function marker BUN (**b**) in the artificial enzymes treated mice. The results were expressed as mean ± SD (*n* = 3 biologically independent animals). Source data are provided as a Source Data file.

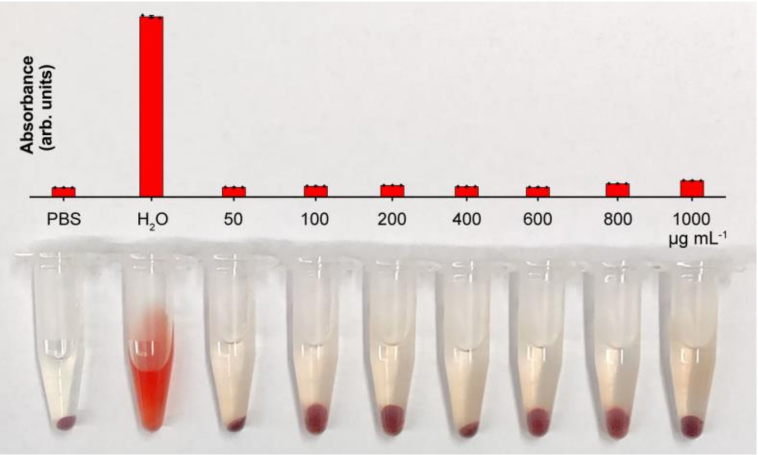

Supplementary Fig. 36: Hemolysis assay of FNA-Fe_3O_4 with different concentrations. PBS and water acted as negative and positive controls, respectively. The results were expressed as mean ± SD (*n* = 3 independent experiments). Source data are provided as a Source Data file.

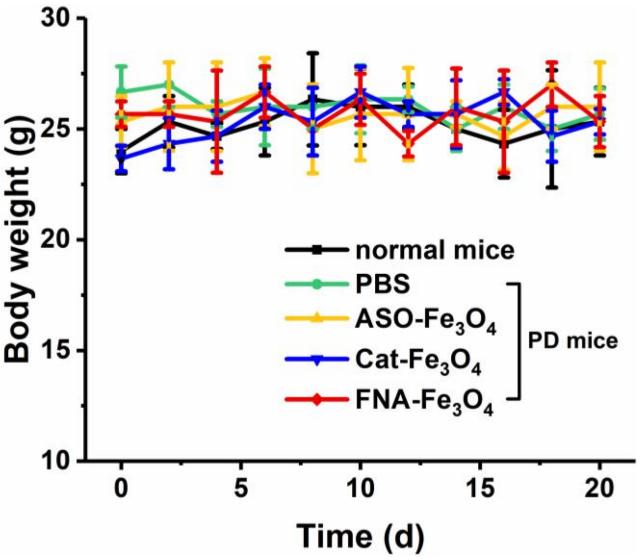

Supplementary Fig. 37: Mice weight growth curves during various treatments. The results were expressed as mean ± SD (n = 3 biologically independent animals). Source data are provided as a Source Data file.
