# Supplementary material for: In situ continuous Dopa supply by responsive artificial enzyme for the treatment of Parkinson’s disease
Source: Nat Commun. 2023 May 9;14:2661. doi: 10.1038/s41467-023-38323-w (PMC10169781; doi:10.1038/s41467-023-38323-w)
Supplement: Supplementary file 2 — Reporting Summary [file 41467_2023_38323_MOESM2_ESM.pdf]

nature portfolio

Corresponding author(s): Chunhua Lu, Huanghao Yang

Last updated by author(s): Apr 23, 2023

Reporting Summary

Nature Portfolio wishes to improve the reproducibility of the work that we publish. This form provides structure for consistency and transparency in reporting. For further information on Nature Portfolio policies, see our Editorial Policies and the Editorial Policy Checklist.

Statistics

For all statistical analyses, confirm that the following items are present in the figure legend, table legend, main text, or Methods section.

n/a	Confirmed	
☐	☒	The exact sample size (n) for each experimental group/condition, given as a discrete number and unit of measurement
☐	☒	A statement on whether measurements were taken from distinct samples or whether the same sample was measured repeatedly
☐	☒	The statistical test(s) used AND whether they are one- or two-sided<br>*Only common tests should be described solely by name; describe more complex techniques in the Methods section.*
☒	☐	A description of all covariates tested
☒	☐	A description of any assumptions or corrections, such as tests of normality and adjustment for multiple comparisons
☐	☒	A full description of the statistical parameters including central tendency (e.g. means) or other basic estimates (e.g. regression coefficient) AND variation (e.g. standard deviation) or associated estimates of uncertainty (e.g. confidence intervals)
☐	☒	For null hypothesis testing, the test statistic (e.g. F, t, r) with confidence intervals, effect sizes, degrees of freedom and P value noted<br>*Give P values as exact values whenever suitable.*
☒	☐	For Bayesian analysis, information on the choice of priors and Markov chain Monte Carlo settings
☒	☐	For hierarchical and complex designs, identification of the appropriate level for tests and full reporting of outcomes
☒	☐	Estimates of effect sizes (e.g. Cohen's d, Pearson's r), indicating how they were calculated

Our web collection on statistics for biologists contains articles on many of the points above.

Software and code

Policy information about availability of computer code

Data collection	TEM images were obtained from FEI Tecnai G2 F20 system operated at 100 keV. XRD analysis was performed using Bruker D8 Advance X-ray diffractometer. ESR spectra data was measured by Bruker A300 spectroscopy. UPLC-MS results were collected from Waters Xevo G2-XS Qtof quadrupole mass spectrometer. HPLC results were collected from Waters e2695 chromatograph. GC-MS results were collected from Shimadzu QP-2020NX chromatograph. The size distributions were measured by Malvern Zetasizer Nano scattering. Flow cytometry data was collected using Beckman Coulter Cytoflex flow cytometer. The cells were imaged through a super resolution confocal laser scanning microscope (Nikon A1+SIM Ti2-E). Cellular fluorescence images were performed on Nikon A1+SIM Ti2-E super resolution confocal laser scanning microscope.
Data analysis	The flowcytometry data of cellular internalization were analyzed on Flowjo software (version 10.0.7). The flowcytometry data of fluorescence imaging by artificial enzymes were analyzed on CytExpert software (version 2.4.0.28, Beckman Coulter Inc.). The mean fluorescence signals from ex vivo organ images were analyzed using Living Image v4.7.2 software. Data statistics and statistical significance calculation was conducted using Microsoft Excel 2016. All results were expressed as mean ± SD. Statistical analyses in all figures were performed by two-tailed Student's t-test. The significance level is *P < 0.05, **P < 0.01 and ***P < 0.001.

For manuscripts utilizing custom algorithms or software that are central to the research but not yet described in published literature, software must be made available to editors and reviewers. We strongly encourage code deposition in a community repository (e.g. GitHub). See the Nature Portfolio guidelines for submitting code & software for further information.

Data

Policy information about availability of data

All manuscripts must include a data availability statement. This statement should provide the following information, where applicable:

- Accession codes, unique identifiers, or web links for publicly available datasets
- A description of any restrictions on data availability
- For clinical datasets or third party data, please ensure that the statement adheres to our policy

All data generated that support the findings of this study are present in the main text and the Supplementary Information file. Source data are provided with this paper.

Human research participants

Policy information about studies involving human research participants and Sex and Gender in Research.

Reporting on sex and gender	N/A
Population characteristics	N/A
Recruitment	N/A
Ethics oversight	N/A

Note that full information on the approval of the study protocol must also be provided in the manuscript.

Field-specific reporting

Please select the one below that is the best fit for your research. If you are not sure, read the appropriate sections before making your selection.

[x] Life sciences [ ] Behavioural & social sciences [ ] Ecological, evolutionary & environmental sciences

For a reference copy of the document with all sections, see nature.com/documents/nr-reporting-summary-flat.pdf

Life sciences study design

All studies must disclose on these points even when the disclosure is negative.

Sample size	No statistical methods were used to predetermine the samle sizes. The sample size (usually n≥3 biologically independent samples) was determined by allowable error, accuracy and resources. The specific sample sizes in the experiments have been indicated in the manuscript. The sample sizes of in vivo experiments (at least three animals in each treatment group) represents the minimum number of animals needed to reach statistical significance ($p<0.05$) between experimental groups. The sample sizes are consistent with those generally adopted and accepted in this field.<br>1. X. Li, et al. Enhanced in Vivo Blood–Brain Barrier Penetration by Circular Tau–Transferrin Receptor Bifunctional Aptamer for Tauopathy Therapy. J. Am. Chem. Soc. 2020, 142(8): 3862–3872.<br>2. W. Feng, et al. 2D vanadium carbide MXenzyme to alleviate ROS-mediated inflammatory and neurodegenerative diseases. Nature Commun. 2021, 12(1): 2203.
Data exclusions	No data were excluded from the analyses.
Replication	For spectroscopy, chromatography, mass spectrometry, catalytic kinetic experiments, individual experiment was independently repeated 3 times. For ex vivo experiments, at least 3 biologically independent samples was used. For in vivo experiments, the displayed data collected from at least 3 biologically independent animals. Every experiments included replicates as described in the Figure legends.
Randomization	All experimental samples and animal models were allocated randomly to each group.
Blinding	In animal experiments, the main investigators were blinded in the process of group allocation and tail vain administration. In other experiments, the main Ivestigators were not blinded since the experimental design, execution and data analysis were performed by the same person.

Reporting for specific materials, systems and methods

We require information from authors about some types of materials, experimental systems and methods used in many studies. Here, indicate whether each material, system or method listed is relevant to your study. If you are not sure if a list item applies to your research, read the appropriate section before selecting a response.

Materials & experimental systems

n/a	Involved in the study
	[x] Antibodies
	[x] Eukaryotic cell lines
[x]	Palaeontology and archaeology
	[x] Animals and other organisms
[x]	Clinical data
[x]	Dual use research of concern

Methods

n/a	Involved in the study
[x]	ChIP-seq
	[x] Flow cytometry
[x]	MRI-based neuroimaging

Antibodies

Antibodies used	1. α-Synuclein monoclonal antibody (Cell Signaling Technology, Cat# 51510, clone number E4U2F, 1:1000 dilution)<br>2. HRP-labeled anti-rabbit secondary antibody (Cell Signaling Technology, Cat# 7074, 1:1000 dilution)<br>3. Tyrosine Hydroxylase rabbit polyclonal antibody (Servicebio, Cat# GB11181, 1:1000 dilution)<br>4. FITC conjugated Goat Anti-Rabbit IgG (H+L) (Servicebio, Cat# GB22303, 1:100 dilution)
Validation	All antibodies were commercially available and were validated by the supplier. All antibodies were used in the study according to the profile of manufacturers. Validation statements are provided on the manufacturer's website.<br>1. α-Synuclein monoclonal antibody (Cell Signaling Technology, Cat# 51510)<br>https://www.cellsignal.com/products/primary-antibodies/a-synuclein-e4u2f-xp-rabbit-mab/51510?site-search-type=Products&N=4294956287&Ntt=e4u2f&fromPage=plp<br>2. HRP-labeled anti-rabbit secondary antibody (Cell Signaling Technology, Cat# 7074)<br>https://www.cellsignal.com/products/secondary-antibodies/anti-rabbit-igg-hrp-linked-antibody/7074<br>3. Tyrosine Hydroxylase rabbit polyclonal antibody (Servicebio, Cat# GB11181)<br>https://www.servicebio.cn/goodsdetail?id=1432<br>4. FITC conjugated Goat Anti-Rabbit IgG (H+L) (Servicebio, Cat# GB22303)<br>https://www.servicebio.cn/goodsdetail?id=259<br>α-Synuclein monoclonal antibody and HRP-labeled anti-rabbit secondary antibody were used in Western Blots.<br>Tyrosine Hydroxylase rabbit polyclonal antibody and FITC conjugated Goat Anti-Rabbit IgG (H+L) were used in Immunofluorescence analysis.

Eukaryotic cell lines

Policy information about cell lines and Sex and Gender in Research

Cell line source(s)	SH-SY5Y and bEnd.3 cell lines were purchased from American Type Culture Collection (cat# CRL-2266 for SH-SY5Y, cat# CRL-2299 for bEnd.3). BV-2 cell line was purchased from Hunan Fenghui Biotechnology Co., Ltd (cat# CL0056 for BV2).
Authentication	American Type Culture Collection and Hunan Fenghui Biotechnology Co., Ltd used morphology, karyotyping, and PCR based approaches to confirm the identity of cell lines and to rule out both intra- and interspeies contamination. Also, the cell lines were frequently checked by their morphological features.
Mycoplasma contamination	All cells were negative for mycoplasma.
Commonly misidentified lines (See ICLAC register)	No commonly misidentified cell line were used.

Animals and other research organisms

Policy information about studies involving animals; ARRIVE guidelines recommended for reporting animal research, and Sex and Gender in Research

Laboratory animals	C57BL/6N (6 months old), and Thy1-SNCA transgenic mice with a C57BL/6N background carrying human wild type SNCA gene driven by the murine thymus cell antigen 1 promoter (6 months old) were purchased from Cyagen Biosciences Co., Ltd. All mice were housed in SPF-grade facilities, cages with standard conditions (50% relative humidity and 12 h light/dark cycle) at 25 °C.
Wild animals	No wild animals were used in this study.
Reporting on sex	The present study did not involve sex-based study design, experiments and results.
Field-collected samples	This study did not involve samples collected from the fields.
Ethics oversight	All animal experiments were monitored and approved by the Institutional Animal Care and Use Committee of Fuzhou University (Approval ID: 2022-SG-014). All mice were kept in accordance with the ethics committee of the National Ministry of Health.

Note that full information on the approval of the study protocol must also be provided in the manuscript.

Flow Cytometry

Plots

Confirm that:

- [x] The axis labels state the marker and fluorochrome used (e.g. CD4-FITC).
- [x] The axis scales are clearly visible. Include numbers along axes only for bottom left plot of group (a 'group' is an analysis of identical markers).
- [x] All plots are contour plots with outliers or pseudocolor plots.
- [x] A numerical value for number of cells or percentage (with statistics) is provided.

Methodology

Sample preparation	SH-SY5Y cells and SNCA-EGFP gene-transfected SH-SY5Y cells were incubated with FNA-Fe3O4 (100 μg mL-1 in DMEM-F12 medium) for 2 h. Afterwards, the cells were washed three times with 1x PBS. Fluorescence images were captured by the CLSM in EGFP and Cy5 channel.
Instrument	Beckman Coulter Cytoflex flow cytometer.
Software	The flowcytometry data of cellular internalization were analyzed on Flowjo software (version 10.0.7).<br>The flowcytometry data of fluorescence imaging by artificial enzymes were analyzed on CytExpert software (version 2.4.0.28, Beckman Coulter Inc.).
Cell population abundance	No sorting was performed.
Gating strategy	Generally, cells were first gated on FSC/SSC. Singlet cells were gated using FSC-H and FSC-A.

- [x] Tick this box to confirm that a figure exemplifying the gating strategy is provided in the Supplementary Information.
