# Supplementary material for: In situ continuous Dopa supply by responsive artificial enzyme for the treatment of Parkinson’s disease
Source: Nat Commun. 2023 May 9;14:2661. doi: 10.1038/s41467-023-38323-w (PMC10169781; doi:10.1038/s41467-023-38323-w)
Supplement: Supplementary file 3 — Description of Additional Supplementary Files [file 41467_2023_38323_MOESM3_ESM.pdf]

Title: Supplementary Video 1
Description: The recorded video in the step-through passive avoidance test.
